# Optimal Lévy-flight foraging in a finite landscape

**DOI:** 10.1098/rsif.2014.1158

**Published:** 2015-03-06

**Authors:** Kun Zhao, Raja Jurdak, Jiajun Liu, David Westcott, Branislav Kusy, Hazel Parry, Philipp Sommer, Adam McKeown

**Affiliations:** 1 CSIRO, Brisbane, Queensland, Australia; 2 CSIRO, Atherton, Queensland, Australia

**Keywords:** Lévy flight, random search, optimal foraging

## Abstract

We present a simple model to study Lévy-flight foraging with a power-law step-size distribution 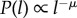 in a finite landscape with countable targets. We find that different optimal foraging strategies characterized by a wide range of power-law exponent *μ*_opt_, from ballistic motion (*μ*_opt_ → 1) to Lévy flight (1 < *μ*_opt_ < 3) to Brownian motion (*μ*_opt_ ≥ 3), may arise in adaptation to the interplay between the termination of foraging, which is regulated by the number of foraging steps, and the environmental context of the landscape, namely the landscape size and number of targets. We further demonstrate that stochastic returning can be another significant factor that affects the foraging efficiency and optimality of foraging strategy. Our study provides a new perspective on Lévy-flight foraging, opens new avenues for investigating the interaction between foraging dynamics and the environment and offers a realistic framework for analysing animal movement patterns from empirical data.

## 1. Introduction

Understanding animal movement is crucial for understanding ecological and evolutionary processes in nature and has a wide range of applications such as ecosystem management, species conservation and disease control [1–5].

In the past decade, it has been widely observed that the movements of many animal species, from albatrosses [6,7] to spider monkeys [8], honeybees [9] to deer [10] and marine predators [11] to human foragers [12,13], appear to exhibit Lévy-flight patterns, i.e. the step-size distribution can be approximated by a power-law 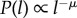 with 1 < *μ* ≤ 3. Despite this apparent similarity, there is an ongoing debate in the scientific community over the existence of Lévy flights in animal movement and the methodology of verifying Lévy flights from empirical data [14–16]. In the meanwhile, scientists, especially theorists, ask the question of why animals do Lévy flight, which fascinates researchers from various disciplines from ecology to physics [17–22].

One common approach to the origin of animal movement patterns is to use the scheme of optimizing random search [23–25]. In a random search model, single or multiple individuals search a landscape to locate targets whose locations are not known *a priori*, which is usually adopted to describe the scenario of animals foraging for food, prey or resources. The locomotion of the individual has a certain degree of freedom which is characterized by a specific search strategy such as a type of random walk and is also subject to other external or internal constraints, such as the environmental context of the landscape or the physical and psychological conditions of the individual. It is assumed that a strategy that optimizes the search efficiency can evolve in response to such constraints on a random search, and the movement is a consequence of the optimization on random search.

A seminal work by Viswanathan *et al.* [26] first studied Lévy-flight foraging through the scheme of optimizing random search. In their model, a forager searches for targets using a random walk with the aforementioned power-law step-size distribution. The forager will keep moving until a target is ‘encountered’, i.e. a target lies within its limited perception range. The search efficiency is defined as the encounter rate of targets, namely the number of visited targets per unit moving distance. The model considers two scenarios: (i) non-destructive foraging, in which the targets are revisitable and (ii) destructive foraging, in which the targets are depleted once visited. For non-destructive foraging, when the targets are sparsely distributed, there exists an optimum exponent *μ*_opt_ ≈ 2 that maximizes the search efficiency, corresponding to the Lévy-flight strategy. For destructive foraging, *μ*_opt_ → 1, corresponding to the ballistic motion, is the optimum solution. It is worth noting that this model only captures an idealized scenario in which learning and prey–predator relationships are ignored [26], and other random search strategies such as intermittent random search can outperform the Lévy-flight strategy in some cases [27].

Recent studies also turn attention to the substantial difference of the value of *μ*_opt_ observed in empirical data. In fact, *μ*_opt_ among different species can range from *μ*_opt_ ≈ 1.59 for human beings [28] to *μ*_opt_ ≈ 2.4 for bigeye tuna [11]. Here *μ*_opt_ also varies significantly among individuals within the same species, e.g. *μ*_opt_ can go from 1.18 to 2.9 in jellyfish [29]. It has been shown that intermediate values of the optimal exponent 1 < *μ*_opt_ ≤ 2 can emerge in the crossover regime between non-destructive and destructive foraging in which targets are regenerated after a period *τ* once depleted [30], or arise in response to landscape heterogeneity [31]. A recent study by Palyulin *et al.* [32] shows that, in the case of searching for a single target in an infinite one-dimensional space, when an external drift (e.g. underwater current, atmospheric wind, etc.) is present, *μ*_opt_ can also vary in the interval 2 ≤ *μ*_opt_ ≤ 3.

In this paper, we propose a simple model to study Lévy-flight foraging in a finite two-dimensional landscape with countable targets. The model considers foraging to be a step-based exploratory search process for distinct targets subject to termination. The forager can revisit the targets and the foraging efficiency is defined as the total number of discovered targets per unit moving distance in this process. We find that different optimal search strategies can emerge in adaption to the interplay between the termination condition and environmental context of the landscape. In particular, different optimum foraging strategies with various exponent *μ*_opt_ can emerge when the termination is regulated by a finite number of steps *N*. In this case, the value of *N*, along with landscape features such as landscape size and number of targets, can play an important role in determining the value of *μ*_opt_. When termination is regulated by a finite moving distance 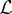, the best strategy is always ballistic motion, corresponding to *μ*_opt_ → 1. To capture more complex foraging dynamics, we also consider stochastic returning (e.g. home-return behaviour) in our model through an exploration–return mechanism [33] and demonstrate that this returning can be another factor that affects the foraging efficiency. Our study not only sheds new light on the understanding of Lévy-flight foraging, but also provides an expanded modelling framework to study animal movement patterns.

## 2. Model

The foraging takes place in a finite two-dimensional *L* × *L*-squared landscape with periodic boundary conditions (when moving across the boundary the forager will come back from the other side of the landscape). There are *K* targets distributed uniformly over the landscape, corresponding to a density *ρ* = *L*^2^/*K*. The forager can detect a target within its perception range *r*_*v*_. The mean free path of the system *λ* is therefore given by *λ* = (2*ρr*_*v*_)*^−^*^1^, which indicates the average straight-line moving distance of detecting or ‘encountering’ a target in the landscape. Without loss of generality, we set *r*_*v*_ = 1, so *λ* = (1/2*ρ*). In this paper, we assume the targets are revisitable, analogous to the case of non-destructive foraging [26]. The goal of the forager is to explore the landscape to find new targets.

**Figure 1.**
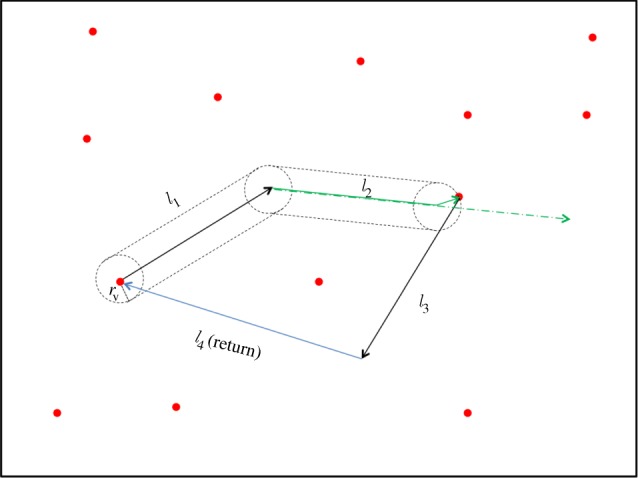
A schematic diagram of the model. This diagram shows a foraging process with *N* = 4 steps. The red dots represent targets. The cylinder formed by black dashed boundaries indicates the detection area during an exploration step. In step one, the forager leaves a target and detects no new target during this step. Note that the forager ignores the departure target. In step two, the forager decides to undertake exploration and detects a target during this step. Therefore, the original probabilistic step (the green dash-dotted line) is truncated to a shorter actual step *l*_2_ (the green solid line). Step three is similar to step one. In step four, the forager decides to return and it flies straight back to the departure target in step one. In a return step, the forager is not attempting to detect targets.

The foraging process is a step-based stochastic process with an exploration–return mechanism. At each step *n*, the forager first decides the type of the step movement: exploration or return. Let us denote the probability of choosing exploration by *p*_*n*_ and the probability of choosing return by *q*_*n*_ = 1 − *p*_*n*_. Moreover, we use *S_n_* to denote the number of distinct targets discovered by the forager up to step *n*, 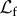 to denote the accumulated moving distance since the forager leaves its last visited target, *l_n_* to denote the moving distance of step *n*, and 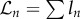 to denote the total moving distance up to step *n*. The foraging movement at step *n* is performed as follows (illustrated in figure 1):

(1) If the decision is exploration, the forager will perform random search in this step. The step-size *l* and the turning angle *θ* are drawn randomly from the pre-defined distribution functions *P*(*l*) and *P*(*θ*). During the step movement, the forager will continuously detect targets. If a target is detected during the step movement, the forager will move to the target in a straight line and the step movement will be truncated. The actual moving distance *l_n_* = *l* − Δ*l* in this case is smaller than the probabilistic moving distance *l* and we set 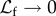. There are two situations of detecting a target. (i) The target is a new target that has not been discovered before and then we update *S_n_* by *S_n_* → *S_n_* + 1 and the location of this new target is memorized by the forager. (ii) The target is a previously visited target. In this case, we do not update *S_n_*. One should note that if this step is the first step the forager leaves a target, the forager will ignore that target in detection to avoid trapping. If no target is detected in this step, we update 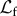 by 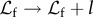.
(2) If the decision is returning, the forager will move to one of the previously visited targets in a straight line. Note that the forager does not attempt to detect targets in a return step, which is analogous to the ‘blind’ phase in intermittent random search [27]. We assume that the forager can memorize the locations of all previously visited targets and randomly decide on the target of the return phase. In this initial model, we focus on this simple approach to modelling memory and leave more complicated memory processes to future work.

Here, we assume that the foraging process starts at a random target in the landscape. That target can be understood as the base of the forager, and its location is recorded in the initial memory of the forager. Therefore, the forager can have at least one location to choose in the return phase. We use a probabilistic function *Θ_n_* ∈ [0, 1] to characterize the termination condition of the process, i.e. the process is terminated at step *n* with probability *Θ_n_*. When a step is performed, we update *n* by *n* → *n* + 1, and we use *N* to denote the total number of steps upon termination.

We then define the search efficiency *η* as the ratio of the total number of distinct targets discovered by the forager to the total moving distance upon termination, which yields2.1
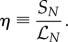


One should note that, besides the stochastic returning in the return phase, the forager can also revisit a previously discovered target if it lies within the forager's perception range in the exploration phase. The revisitation during exploration may occur in two scenario: (i) the forager taking advantage of the chance proximity of a previously discovered target to relieve movement constraints (e.g. to rest or supplement energy) prior to reinitiating exploration and (ii) the forager can detect a target within its perception range, but is not able to identify the resource availability in the detected target without a revisitation. In such context, the target can be understood to be a site that contains resource (e.g. a tree with fruits). The random search dynamics in the exploration phase in our model resembles the non-destructive foraging in [26], while the definition of search efficiency that only accounts for distinct targets discovered by the forager resembles the case of destructive foraging with exploitation.

Finally, we specify the random search dynamics and the exploration–return mechanism. In this paper, we use a power-law step-size distribution for the random search, which yields2.2
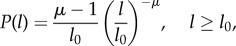
where 1 < *μ* ≤ 3 is the power-law exponent which serves as a control parameter of the random search foraging strategy. The lower bound *l*_0_ represents the natural limit of step-size. The movement converges to the Gaussian (Brownian motion) when *μ* ≥ 3, and to ballistic motion when *μ* → 1. For simplicity in this paper, we set *l*_0_ = *r_v_* = 1 and we use a uniform distribution *P*(*θ*) = 1/2*π* for the turning angle.

To specify *p*_*n*_ and *q*_*n*_, we assume that the forager makes a decision between exploration and return according the following rule: *the longer it has travelled since it leaves the last visited target, the more likely it will decide to return*. The decision rule here reflects the accumulated resistance on the forager's will for exploration as the moving distance increases, such as the decline of energy level, accumulated stress of finding no new targets or other behavioural regularities. Therefore, *p*_*n*_ can be represented by a non-increasing function of 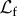. Specifically in this paper, we define *p*_*n*_ as an exponential function2.3
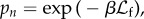
where *β* is a control parameter for tuning the intensity of such returning. A higher *β* leads to a higher likelihood of stochastic returning during exploration, which models more conservative foraging behaviour. Similar dynamics have been observed empirically in both animals [34] and humans [35]. The stochastic returning can be used to model the home-return patterns [33] or the central-place foraging dynamics [36,37]. When *β* = 0 such that *p*_*n*_ = 1, the model is without a return phase and the foraging dynamics resembles the non-destructive foraging in [26].

In summary, the foraging in our model is tuned by five parameters, namely *μ*, *N*, *L*, *K* and *β*. The power-law exponent *μ* characterizes the random search strategy. The landscape size *L* and the number of targets *K* represent the environmental features. The number of steps *N* regulated by the probabilistic function *Θ_n_* specifies the termination of the foraging. The intensity of stochastic returning *β* is an additional parameter to characterize more complex foraging dynamics.

## 3. Results and discussions

### 3.1. The case with *β* = 0

We first discuss the simple case without the stochastic returning by setting *β* = 0 and *p*_*n*_ = 1. To gain better insight into the model, we derive a closed-form expression for the search efficiency *η* using mean-field approximation. We then investigate the relationship between *η*(*μ*) and other parameters of the model using a combination of numerical simulations and analytical evaluation.

#### 3.1.1. Approximate mean-field solution

**Figure 2.**
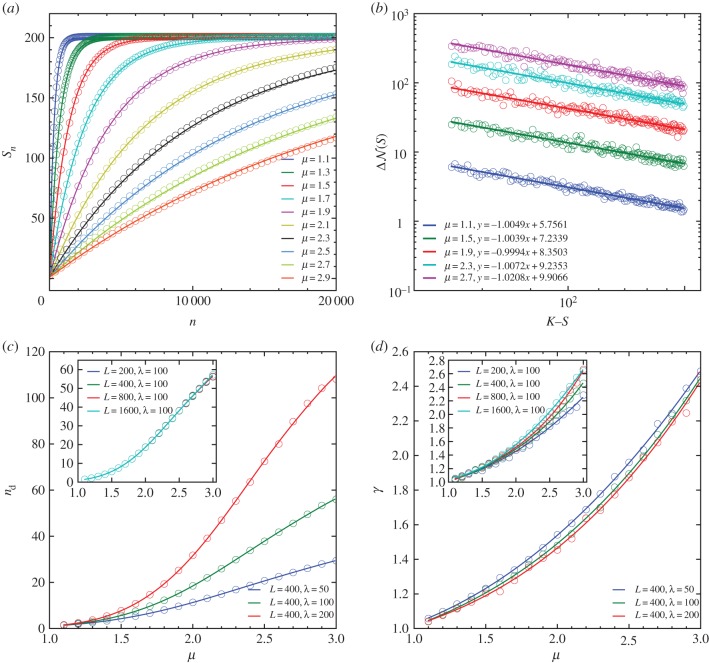
(*a*) The number of discovered targets *S_n_* versus step number *n*. The curves in different colours from top to bottom correspond to various *μ* ∈ [1.1, 2.5] with an interval of 0.2. Here, we use the landscape size *L* = 200 and the number of targets *K* = 200. The dots represent simulation results averaged for 100 realizations, and the solid lines represent the corresponding mean-field solution given by equation (3.3). (*b*) The mean number of steps between the discovery of the *S*th target and the (*S* + 1)th target 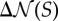 versus the number of undiscovered targets *K* − *S*. The dots represent simulation results, and the lines represent the corresponding linear regression fit. (*c*) The mean number of steps between two consecutive detections *n*_d_ as a function of *μ* for different values of *L* and *K*. Here, *K* is adjusted to obtain the corresponding *λ* given *L*. The solid lines represent the nonlinear fitting 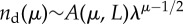. (*d*) The constant coefficient *γ* as a function of *μ* for different values of *L* and *K*. The solid lines represent the cubic polynomial fitting.

To evaluate equation (2.1), we first calculate the numerator *S*_*N*_. We denote by 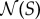 the mean number of steps from the beginning to the discovery of the *S*th new target, and 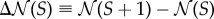 the mean number of steps between the discovery of the *S*th target and the (*S* + 1)th target. Intuitively, since the foraging is confined in a finite landscape with limited number of targets, as the forager explores the landscape more, the number of undiscovered targets decreases and discovering new targets becomes more difficult. If the forager detects a target at step *n*, the probability that the detected target is new, denoted by *p*_new_, should be approximately proportional to the number of undiscovered targets at step *n*, namely *p*_new_ ≈ (*K* − *S*_*n*_)/*γK*, where *γ* is a constant coefficient. Therefore, to discover one more new target, the forager has to make 1/*p*_new_ detections on average, which gives3.1
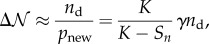
where *n*_d_ is the mean number of steps between two consecutive detections. The assumption that 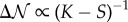 indicated by equation (3.1) is supported by the numerical simulation, as shown in figure 2*b*. The increment of *S_n_* at each step *n* can be written as follows:3.2
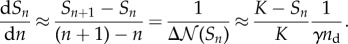
The above differential equation can be solved with initial condition *S*_0_ = 1, which yields3.3
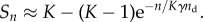

Then we calculate the denominator 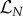. In mean-field approximation, 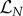 can be simply expressed as 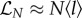, where 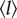 is the mean step-size given by [26]3.4
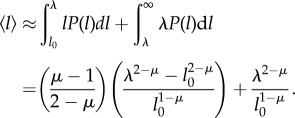


The above equation captures well the simulation results, as shown in figure 7*b*. Via equations (3.3) and (3.4), we are able to calculate the search efficiency *η*.

To evaluate *S*_*n*_ analytically through equation (3.3), we still need to know *n*_d_ and *γ*. Unfortunately, a closed-form of *n*_d_ and *γ* in equation (3.3) remains mathematically elusive, so we apply curve fitting to the simulations curves of *S*_*n*_ to obtain the value of *C* = *γn*_d_ in equation (3.3). Consequently, we can estimate *γ* using *γ* = *C*/*n*_d_, where *n*_d_ is obtained from simulation. The advantage of this approach is that both *γ* and *n*_d_ remain approximately constant as *n* increases. Therefore, we can obtain the value of *C* by performing the simulation up to a small number of steps (e.g. up to 20% targets in the landscape have been discovered in our study), and then use the analytical solution to extrapolate the results for large *n. S*_*n*_ evaluated in equation (3.3) with a fitting parameter *C* is in good agreement with the numerical simulation, as shown in figure 2*a*. With *L* and *K* given (*λ* is determined), we can also use curve fitting to find *γ*(*μ*) and *n*_d_(*μ*) by performing numerical simulations for a small number of discrete values of *μ*, as shown in figure 2*c,d*. In particular, for a given *λ* , we use a cubic polynomial function to approximate *γ*(*μ*), and an exponential function 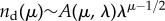 [26,38] to approximate *n*_d_(*μ*), where *A*(*μ*, *λ*) is a fifth degree polynomial function of *μ* determined by polynomial regression. Our results also suggest that, with *μ* given, *γ* depends on both *L* and *K*, while *n*_d_ only depends on *λ*, as shown in figure 2*c,d*.

We note that both the denominator and numerator in equation (2.1), denoting the total moving distance 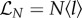 and total number of discovered targets *S_N_*, respectively, are a decreasing function of *μ*. Intuitively, this is easy to understand. In an N-step truncated Lévy flight, decreasing *μ* not only leads to a larger mean step-size as indicated by equation (3.4), but also enlarges the searched area so that more new targets can be discovered by the forager. On the other hand, *S_N_* and *L_N_* are also increasing functions of *N*, i.e. termination upon larger number of steps leads to more discovered targets and longer moving distances. In the following, we study how the foraging efficiency and optimality of search strategy depend on the number of steps *N*, the landscape size *L* and the number of targets *K*.

#### 3.1.2. Number of steps *N*

**Figure 3.**
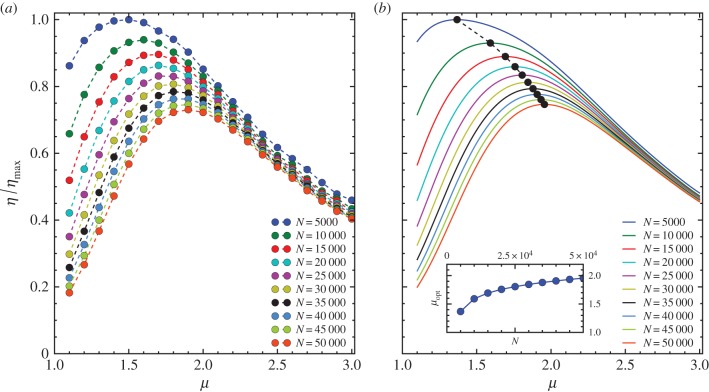
(*a*) The rescaled search efficiency *η*/*η*_max_ versus the power-law exponent *μ* for different values of the total number of steps *N* from numerical simulation with the intensity of stochastic returning *β* = 0, the landscape size *L* = 1000, the number of targets *K* = 5000 and the termination condition *Θ_n_* = *δ*(*n* − *N*). The results are averaged over 100 realizations. The rescaling factor *η*_max_ = *η*(*μ* = *μ*_opt_, *N* = 5000) is the overall maximum search efficiency. (*b*) *η*/*η*_max_ versus *μ* from mean-field calculation. The black dots indicate the peaks of the curves. The inset of panel (*b*) shows *μ*_opt_ versus *N*. The curves in panels (*a*,*b*) with different colours from top to bottom correspond to various *N* ∈ [5 × 10^3^, 5 × 10^4^] with an interval of 5000. Note that the discrepancy between the simulation and the mean-field approach here is mostly due to the slight deviation of equation (3.4), as shown in figure 7*b*. This can be improved by better calibrating the form of equation (3.4), e.g. using curve fitting as we obtain the form of continuous function for *n*_d_(*μ*) and *γ*(*μ*).

To study the influence of the number of steps *N*, we use a probabilistic function *Θ_n_* = *δ*(*n* − *N*) to terminate the foraging process at *n* = *N* step for any given value of *μ*. Here *δ*(*·*) is the Kronecker delta function, i.e. *δ*(*x*) = 0 if *x* = 0 and *δ*(*x*) = 1 if *x ≠* 0. We perform numerical simulation with *L* = 10^3^, *K* = 5000 and *λ* = 100. The search efficiency *η* is then fully determined by the number of foraging steps *N* and the power-law exponent *μ*, which yields *η* = *η*(*μ*, *N*). Surprisingly, for any given value of *N*, there exists an optimal exponent *μ*_opt_ which maximizes *η*. We find that *μ*_opt_ shifts substantially as *N* varies and is overall an increasing function of *N*, as shown in figure 3*a*. The corresponding *η*(*μ*, *N*) calculated by the mean-field approach using equations (3.2) and (3.3), as shown in figure 3*b*, align with the results from numerical simulation in figure 3*a*.

A simple explanation for the presence of such optimality relates to the increased difficulty in discovering new targets in the later stage of the foraging process, particularly for smaller *μ*. Recall that the forager can discover new targets more rapidly in the beginning (high-efficiency stage) but then enters into a difficult stage for discovering new targets in the latter steps (low-efficiency stage) as the number of new targets drops. The random search with smaller *μ* will enter this low-efficiency stage earlier in the foraging process, as shown in figure 2*a*. The gain in *S_N_* from reducing *μ* will decline steadily, and will eventually no longer compensate for increases in 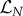; at that point, *η* reaches its maximum.

Here *μ*_opt_ as a function of *N* can be obtained by numerically solving 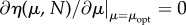 using the continuous *η*(*μ*, *N*) from the mean-field approach, as shown in the inset of figure 3*b*. We note that when *N* → 1 the optimal exponent will approach *μ*_opt_ → 1 approximately corresponding to ballistic motion. The optimal exponent can also approach *μ* = 3 for a sufficiently large *N*, which means the optimal random search can go through a transition from Lévy flight (1 < *μ* < 3) to Brownian motion (*μ* ≥ 3) as *N* increases.

#### 3.1.3. Landscape size *L* and number of targets *K*

**Figure 4.**
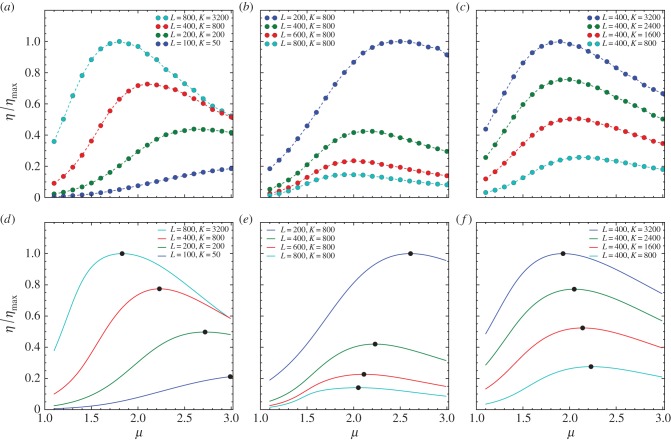
Panels (*a–c*) show the rescaled search efficiency *η*/*η*_max_ versus the power-law exponent *μ* for different values of the landscape size *L* and the number of targets *K* from numerical simulation with the intensity of stochastic returning *β* = 0 and the termination condition *Θ_n_* = *δ*(*n* − 20 000). The results are averaged over 100 realizations. The curves in panel (*a*) from bottom to top correspond to (*L*, *K*) = (100, 50), (200, 200), (400, 800), (800, 3200) such that the mean free path *λ* = 100 remains constant. The curves in panel (*b*) from top to bottom correspond to *L* = 200, 400, 600, 800 with *K* = 800 kept constant. The curves in panel (*c*) from top to bottom correspond to *K* = 800, 1600, 2400, 3200 with *L* = 400 kept constant. Panels (*d–f*) show *η*/*η*_max_ evaluated by the mean-field solution, corresponding to the results from numerical simulation in panels (*a–c*), respectively. The black dots indicate the peak of the curves.

We then study how the foraging efficiency depends on the landscape size *L* and number of targets *K* under the termination condition *Θ_n_* = *δ*(*n* − *N*). We evaluate the search efficiency by varying the landscape size *L* and the number of targets *K* in three different combinations: (1) varying *L* and *K* simultaneously with the mean free path *λ* = *L*^2^/2*K* kept constant; (2) varying *L* with *K* kept constant and (3) varying *K* with *L* kept constant. As shown in figure 4, the presence of optimality in *η*(*μ*) still remains but *μ*_opt_ varies for different combinations of *L* and *K*.

Intuitively, with *N* and *λ* given, as *K* increases, the optimal exponent *μ*_opt_ will shift to a smaller value since the foraging can stay in the high-efficiency stage for longer total moving distances, accounting for the situation in combination (1). Similarly, with *N* and *K* given, as *λ* decreases, *μ*_opt_ will shift to a larger value, since the average distance for discovering new targets becomes shorter and the low-efficiency stage comes earlier for smaller *μ*, accounting for the situation in combination (2). The coupled effect of increasing *K* and decreasing *λ* is studied through combination (3). In this case, as *K* increases, *μ*_opt_ still shifts to a smaller value, but the shifting is not as significant as the case in combination (1).

The results here suggest that the foraging efficiency and optimal foraging strategy characterized by *μ*_opt_ can be highly sensitive to environmental context and landscape features under some termination conditions.

#### 3.1.4. Termination condition *Θ_n_*

The foraging defined in our model is considered to be an individual search process in a finite area subject to termination. This setting can account for two scenarios in the real-world: (1) foraging within a restricted area during a certain foraging season; (2) foraging in a fractal landscape (where resources are clustered in patches) such that searching each patchy area can be viewed as a single process. In scenario (2), owing to the depletion of new targets, the forager has to decide on when to terminate the foraging. The probabilistic function *Θ_n_*, which characterizes the termination condition and regulates the number of steps *N*, can reflect the animal's prior experience, its physical condition or behavioural regularity, or other environmental conditions such as seasonal variation that can have an influence on the forager's decision to terminate foraging/change foraging area. In this context, various optimal foraging strategies may evolve in response to different termination conditions coupled with the environmental context of the foraging area to achieve the highest search efficiency.

**Figure 5.**
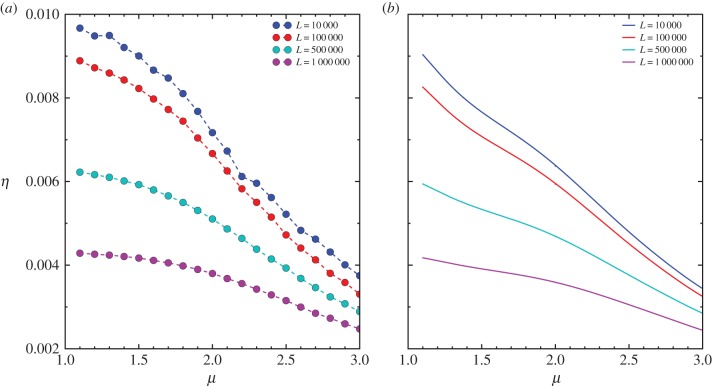
(*a*) The search efficiency *η* versus the power-law exponent *μ* for different values of the threshold moving distance 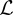 from numerical simulation with the intensity of stochastic returning *β* = 0, the landscape size *L* = 1000, the number of targets *K* = 5000 and the termination condition 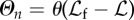 where 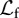 is the accumulated moving distance. The results are averaged over 100 realizations. (*b*) *η* evaluated by the mean-field solution by setting the total number of steps *N* by 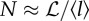 in equation (3.3).

The results displayed in figures 3 and 4 present the optimality of foraging strategy for the special case with *Θ_n_* = *δ*(*n* − *N*) in which the termination is completely regulated by a finite number of steps. For comparison, we study another special case with a termination condition 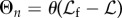, where *θ*(·) is the step function such that *θ*(*x*) = 0 if *x* < 0 and *θ*(*x*) = 1 if *x* ≥ 0. In this case, foraging is terminated once the accumulated moving distance 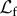 exceeds some threshold 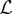, and the best strategy is always ballistic motion corresponding to *μ*_opt_ → 1, as shown in figure 5. The results here imply that, if the forager always prefers exploring the landscape with a prefixed length of moving distance 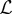 (or a certain amount of energy for exploration if we consider energy expenditure is proportional to 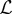), the optimal search strategy will evolve to ballistic motion regardless of the choice of 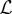. On the other hand, if the forager explores the landscape with a random moving distance 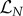 (or a random amount of energy) regulated by a certain number of steps *N*, various optimal search strategies from ballistic motion (*μ* → 1) to Lévy flight (1 < *μ* < 3) to Brownian motion (*μ* ≥ 3) can emerge depending on landscape features. This situation is more likely to occur in scenario (1), in which the animal may be subject to daily regularity during the foraging season. For instance, the animal may only perform a regular number of foraging steps (trips/bouts) per day, and the moving distance in each step is randomly distributed. It is also interesting to note that the optimality under the termination condition *Θ_n_* = *δ*(*n* − *N*) can emerge before the landscape has been fully exploited. Therefore, the optimality we observe here remains valid even if we introduce an additional condition to the termination, i.e. *Θ_n_* = *δ*(*n* − *N*) + *δ*(*S_n_* − *K*), such that the foraging will be terminated once the forager discovers all targets (or we assume that the forager has prior knowledge of the number of targets) and will not fall into the ‘zero-gain’ regime.

The two special cases discussed above demonstrate that different optimalities of foraging strategy can exist under alternative termination conditions. Generally speaking, termination can be triggered by more complex mechanisms, and *Θ_n_* can be a function of multiple variables such as *N*, 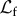, 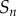 and other observable or hidden quantities. The optimality should be considered subject to the detailed termination mechanism.

### 3.2. The general case with *β* > 0

**Figure 6.**
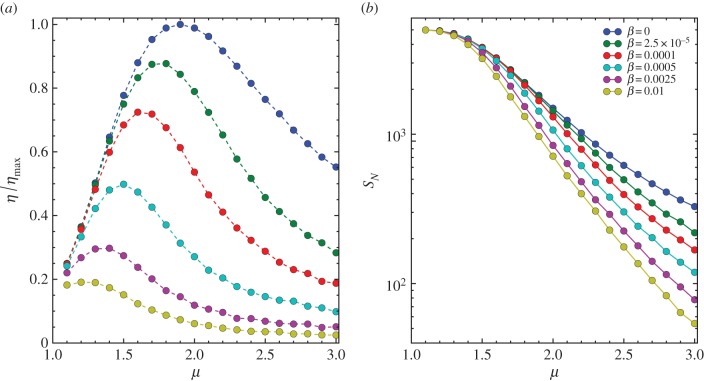
(*a*) *η*/*η*_max_ versus *μ* for various intensities of stochastic returning *β* from numerical simulation with the landscape size *L* = 1000, the number of targets *K* = 5000 and the termination condition *Θ_n_* = *δ*(*n* − 50 000). The results are averaged over 100 realizations. The dots represent simulation results, and the dashed lines are a guide to the eye. (*b*) The total number of discovered targets *S_N_* versus *μ*. The curves in panels (*a*,*b*) with different colours from top to bottom corresponding to various *β* with increasing values as indicated in the legend of panel (*b*).

We now turn our attention to the case with *β* > 0 in which stochastic returning is present. We briefly study this case with the termination condition *Θ_n_* = *δ*(*n* − *N*). It would be interesting to study other stochastic returning mechanisms and their interplay with the termination decision in future. We perform numerical simulation with various intensity *β* and exponent *μ*, as shown in figure 6*a*. As might be expected, *β* has a significant impact on the search efficiency. When *β* increases, the optimal exponent *μ*_opt_ will shift to a smaller value.

The result here indicates that an optimal search strategy can be tuned by the stochastic returning which is widely observed in animals. One should note that the stochastic returning here is an additional constraint for the random search, which can be associated with a wide range of intrinsic physical or psychological features of the forager or external environmental context of the landscape. For example, a high value of *β* can represent a harsh foraging environment that drives the forager to return more frequently. A higher intensity of stochastic return leads to lower gain (fewer targets discovered) and higher cost (longer moving distance) in foraging and is hence harmful to the search efficiency.

**Figure 7.**
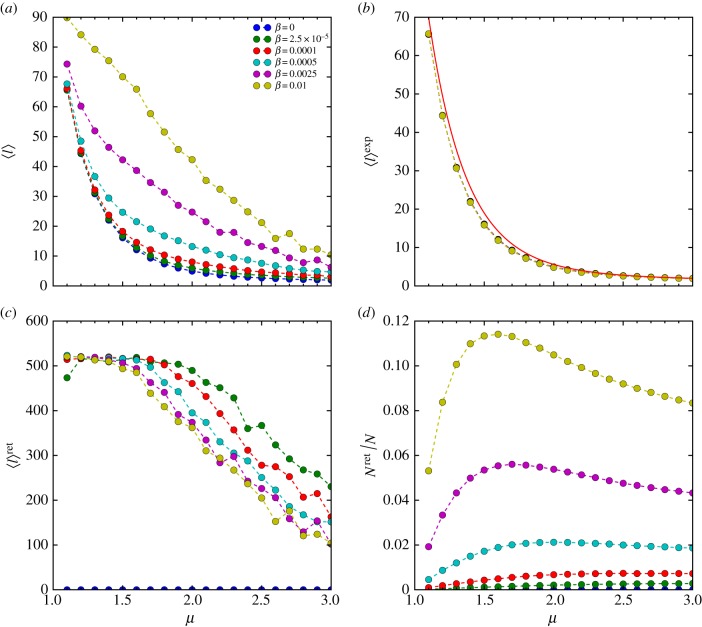
(*a*) The mean step-size 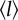 versus the power-law exponent *μ*. (*b*) The mean step-size in exploration 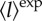 versus *μ*. The red solid line represents equation (3.4). (*c*) The mean step-size in return 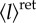 versus *μ*. (*d*) The ratio of the number of return steps *N*^ret^ to the total number of steps *N* versus *μ*. In panels (*a–d*), the dots represent simulations results, and the dashed lines are a guide to the eye. Different colours correspond to different values of *β*, as indicated in the legend of panel (*a*). The results are obtained from numerical simulation with the landscape size *L* = 1000, the number of targets *K* = 5000 and the termination condition *Θ_n_* = *δ*(*n* − 50 000) and are averaged over 100 realizations.

**Figure 8.**
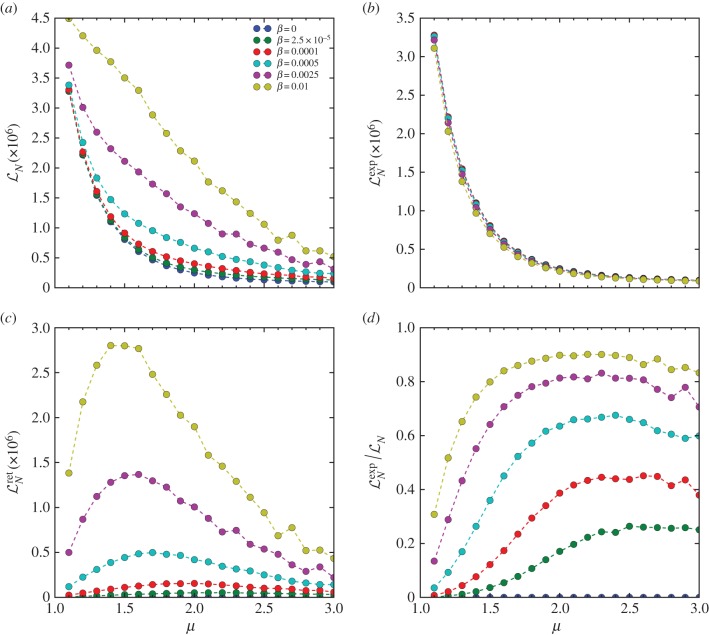
(*a*) The total moving distance 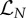 versus the power-law exponent *μ*. (*b*) The total moving distance in exploration 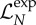 versus *μ*. (*c*) The total moving distance in return 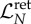 versus *μ*. (*d*) The ratio of 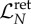 to the total number of steps 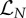 versus *μ*. In panels (*a–d*), the dots represent simulations results and the dashed lines are a guide to the eye. Different colours correspond to different values of *β*, as indicated in the legend of panel (*a*). The results are obtained from numerical simulation with the landscape size *L* = 1000, the number of targets *K* = 5000 and the termination condition *Θ_n_* = *δ*(*n* − 50 000) and are averaged over 100 realizations.

Finally, we discuss some interesting characteristics regarding 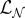 under the current stochastic returning mechanism. In the presence of stochastic returning, 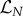 is composed of the moving distance of the exploration and the return phase, respectively, namely 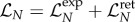, where the superscript denotes the corresponding phase. In particular, we can write 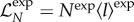 and 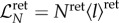 with 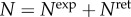, where the superscripted *N* and 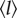 denote the number of steps and mean step-size in each phase. These quantities as a function of *μ* and *β* are shown in figures 7 and 8. Interestingly, we find that 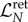, as well as the portion of return steps 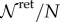, are not a monotonic function of *μ* and exhibit a maximum. A possible explanation for this peak is that the value of *μ* determines the balance between the stochastic returning and accidental rediscovery of a known target. A lower *μ* results in longer steps, leading to higher likelihood of stochastic returning. On the other hand, a higher *μ* results in shorter steps, leading the forager to revisit the same target many times consecutively by the accidental rediscovery (the trapping phenomenon in the model). Following this reasoning, the peaks in 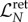 may reflect the value of *μ* that maximizes the aggregated return distance due to the stochastic and accidental returns. Fully understanding these phenomena is beyond the scope of this paper.

## 4. Conclusion

In this paper, we have presented a simple model to study Lévy-flight foraging in a finite landscape with countable targets. The first important message from our results is that the interplay between the termination of foraging and environmental features, such as the landscape size and target density, can have a significant influence on the development of an optimal foraging strategy. This is in contrast to most previous models which are not sensitive to termination condition, such as the ‘non-destructive’ truncated Lévy-flight foraging [26] or other random search models that focus on the search for a single target [32]. The termination condition in our model can be used to account for the decision-making mechanism that drives the animal to stop foraging in a certain area. In particular, when the number of steps (or trips/bouts in foraging) plays a role in the termination condition, a wide range of *μ*_opt_, which captures foraging dynamics from ballistic motion (*μ*_opt_ → 1) to Lévy flight (1 < *μ*_opt_ < 3) to Brownian motion (*μ*_opt_ ≥ 3), can evolve in response to other constraints such as the landscape size, number of targets as well as stochastic returning (e.g. home-return behaviour).

This idealized model allows a variety of future extensions. In the current approach, we do not consider energy intake and consumption, and simply assume that the forager always has enough energy to perform long flights. One important improvement to the model is to incorporate an adaptive foraging strategy based on available energy. As a supplement to this work, one can also study other termination conditions to capture more complex internal regularity, memory mechanisms or decision-making of foraging [39–43], as well as the coevolution of the foraging dynamics and termination mechanism. Moreover, one can consider time-variant and heterogeneous distribution of targets in the landscape, which can lead to a study of how the foraging strategy adapts to the environmental changes in the presence of cognition and memory. It is also worth extending the model to incorporate multiple foragers and studying the collective process of competition and cooperation in foraging [44].

It would be interesting for researchers to test our model in empirical data. One direct observable consequence suggested by our study is that the value of *μ*_opt_ may have a relationship with some dependent quantities such as the total moving distance (energy expenditure) or the number of steps (trips/bouts) of a foraging process, as well as the size or the resource distribution of the foraging area. This dependence may be tested by measuring the correlation between the value of *μ*_opt_ and these quantities among individuals. Finally, although it is initially proposed to study animal foraging, our model can be used generally in other random search or biological encountering processes as well.
